# The Role of NF-κB in Endometrial Diseases in Humans and Animals: A Review

**DOI:** 10.3390/ijms24032901

**Published:** 2023-02-02

**Authors:** Łukasz Zdrojkowski, Tomasz Jasiński, Graça Ferreira-Dias, Bartosz Pawliński, Małgorzata Domino

**Affiliations:** 1 Department of Large Animal Diseases and Clinic, Institute of Veterinary Medicine, Warsaw University of Life Sciences, 02-787 Warsaw, Poland; 2 CIISA-Center for Interdisciplinary Research in Animal Health, Faculty of Veterinary Medicine, University of Lisbon, 1300-477 Lisbon, Portugal; 3 Associate Laboratory for Animal and Veterinary Sciences (AL4AnimalS), 1300-477 Lisbon, Portugal

**Keywords:** NF-κB, endometritis, endometriosis, endometrosis, comparative medicine

## Abstract

The expression of genes of various proinflammatory chemokines and cytokines is controlled, among others, by the signaling pathway of the nuclear factor kappaB (NF-κB) superfamily of proteins, providing an impact on immune system functioning. The present review addresses the influence and role of the NF-κB pathway in the development and progression of most vital endometrial diseases in human and animal species. Immune modulation by NF-κB in endometritis, endometrosis, endometriosis, and carcinoma results in changes in cell migration, proliferation, and inflammation intensity in both the stroma and epithelium. In endometrial cells, the NF-κB signaling pathway may be activated by multiple stimuli, such as bacterial parts, cytokines, or hormones binding to specific receptors. The dysregulation of the immune system in response to NF-κB involves aberrant production of chemokines and cytokines, which plays a role in endometritis, endometriosis, endometrosis, and endometrial carcinoma. However, estrogen and progesterone influence on the reproductive tract always plays a major role in its regulation. Thus, sex hormones cannot be overlooked in endometrial disease physiopathology. While immune system dysregulation seems to be NF-κB-dependent, the hormone-independent and hormone-dependent regulation of NF-κB signaling in the endometrium should be considered in future studies. Future goals in this research should be a step up into clinical trials with compounds affecting NF-κB as treatment for endometrial diseases.

## 1. Introduction

The nuclear factor kappaB (NF-κB) is one of the transcription factors responsible for cellular chemoresistance [1]. The NF-κB signaling pathway controls the expression of genes of proinflammatory chemokines and cytokines, adhesion molecules, chemoattractants for inflammatory cells, and antigen receptors on immune cells [2]. It remains a critical regulator of immune systems in most multicellular organisms. It can be found in most animal cell types, involved in multiple cellular responses [3,4,5]. Proinflammatory chemokines and cytokines act under both physiological conditions and pathological processes, and their aberrant production leads to immune system dysregulation, which plays a role in some endometrial diseases’ initiation and progression [6]. In the case of endometritis, NF-κB is strongly involved in the activation of proinflammatory genes which are critical for uterine response to infection and inflammation [7]. In the case of endometriosis, NF-κB stimulates the expression of genes that regulate endometriotic cell adhesion, migration, and proliferation, as well as extracellular matrix (ECM) remodeling and inflammation intensity in ectopic endometrium [1,6], whereas in the case of endometrosis, NF-κB seems to take part in the mediation of the expression of genes that stimulate proinflammatory chemokines and inhibit anti-inflammatory cytokine in fibrotic endometrium, as well as stimulating ECM remodeling and chemotaxis [8,9]. Moreover, NF-κB is also associated with chronic inflammation in neoplastic development, as well as with the mediation of insensitivity to growth inhibitory signals, avoidance of apoptosis, angiogenesis, and metastasis [10]. As the endometrium undergoes cyclic changes in all species, its cells have to be constantly supplied with signal molecules, which can lead to increased susceptibility to actions of the NF-κB pathway [11].

Animal models are used widely in studies regarding the NF-κB pathway, especially using genetically modified mice. Especially in cancer and chronic inflammatory diseases it is an important object of interest, with the possible development of new therapeutic approaches [5]. Additionally, in endometriosis studies mice and rats are being used [1]. While the prevalence and importance of endometrial diseases varies between species, most of the mechanisms leading to their development are alike [4,5]. This indicates that we can learn about the NF-κB pathway in endometrial diseases on both sides—using animal data to improve human studies, and using human data in animal research.

In this review, the role of NF-κB in the pathogenesis of endometrial diseases in humans and animals is described. As NF-κB signaling has a close relationship with estrogen and progesterone, which are the key regulatory factors for the initiation and progression of endometrial diseases, the hormone-dependent regulation of NF-κB pathways is also summarized.

## 2. NF-κB Signaling in Endometrial Disease

Many groups have reported that the activation of NF-κB plays, through complex mechanisms [12,13,14,15,16], a vital role in the regulation of the initiation and progression of many endometrial diseases such as endometritis [7,17,18,19], endometriosis [6,19,20], endometrosis [8,9,11], and endometrial carcinoma [16,21,22,23]. As the referred endometrial diseases have a marked impact on reproductive health, causing subfertility and infertility, the selected recent research on pathways, targets, and mechanisms are summarized in Table 1. 

### 2.1. NF-κBa Activation

The superfamily of NF-κB proteins consists of five known members: protein RelA of NF-κB superfamily (RelA (p65)), protein RelB of NF-κB superfamily (RelB), protein cRel of NF-κB superfamily (cRel), protein NF-κB1 of NF-κB superfamily (NF-κB1 (p50/p105)), and protein NF-κB2 (NF-κB2 (p52/p100)) [11]. Each of the five NF-κB members interacts with suitable inhibitory factors belonging to the family of inhibitors of κB (IκB) (IκBα, IκBβ, IκBε, or Bcl-3) or the C-terminal sequences of the NF-κB precursor proteins (p105 and p100) [12]. The RelA, RelB, and cRel proteins share a homology domain Rel, which is a transcription activation domain, allowing control of the transcription of DNA molecules, whereas NF-κB1 and NF-κB2 proteins are precursor proteins, which need proteolytic activation and forming dimers with suitable Rel protein [13]. Heterodimers of NF-κB are present in the cytoplasm bound to IκB proteins, which are phosphorylated by IκB kinases (IKKs) when NF-κB signaling is activated [1]. This activation takes place through canonical and noncanonical signaling pathways, which engage RelA/NF-κB1 and RelB/NF-κB2 subunits, respectively [15]. However, regardless of the pathway, this IκB-related activation by the IKK complex allows NF-κB dimers to perform nuclear translocation and activation of the transcription of target genes [1,16].

In endometrial cells, both stroma and epithelium, the NF-κB signaling pathway may be activated by multiple stimuli, such as bacterial parts, cytokines, or hormones binding to specific receptors [1,17,22]. Each stimulus may activate the IKK complex, which phosphorylates IκB proteins and thus allows the cytoplasmic heterodimer to be released and translocated to the nucleus [25] on a canonical (RelA/NF-κB1) [7,8,9,16,18,19,21,22] or noncanonical (RelB/NF-κB2) [8,9,16] pathway. This translocation of a transcription activation domain elicits the transcriptional activity of genes [1] of several receptors, enzymes, cytokines/chemokines, and other signaling proteins, indicating the key role of NF-κB signaling in the pathogenesis of the most common endometrial diseases [6,7,8,9,16,17,18,19,20,21,22,23,24]. In this way, NF-κB signaling can regulate the endometrial cells’ reaction to infection and inflammation [7]; immune cells’ chemotaxis [6]; cellular behaviors of endometrial cells including cells proliferation, adhesion, migration [6], apoptosis [21], and invasion [23]; as well as ECM remodeling [19], which is illustrated in detail in the following subsections.

### 2.2. Endometritis

Endometrial infections are the most common reproductive diseases in dairy cows [4,26] and breeding mares [27,28], whereas the clinical significance of chronic endometritis has rarely been a concern in human clinical practice [29]. In the course of endometritis, the infiltration of immune cells into the endometrial stroma is observed, while the type and intensity of infiltration varies between the subtype of endometritis [29,30]. Suppurative endometritis, which is mostly caused by a bacterial infection, is characterized by the recruitment of neutrophils and T cells, whereas nonsuppurative endometritis is characterized by the infiltration of lymphocytes and plasma cells [30], while macrophages, eosinophils [28], and mast cells [31] are uncommon. In response to the intrauterine bacterial infection, Toll-like receptor 4 (TLR4) on the endometrial cells of luminal epithelium recognizes lipopolysaccharide (LPS) derived from Gram-negative bacteria and activates NF-κB and mitogen-activated protein kinase (MAPK) pathways [7]. 

**Figure 1:**
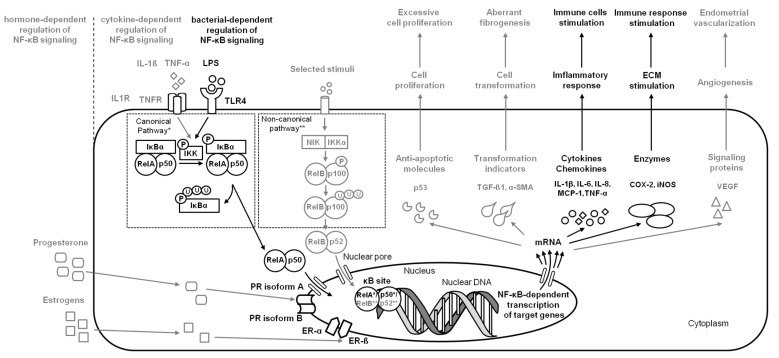
A simplified schema of the NF-κB signaling pathway in the endometrium in the case of endometritis. Studied pathways are marked in black. Pathways requiring further research are marked in grey. Both canonical pathway * and noncanonical pathway ** are considered.

In human [17] and bovine endometrium [7,18], activation of these pathways stimulates the release of interleukins (ILs): IL-1β, IL-6, and IL-8, as well as monocyte chemoattractant protein 1 (MCP-1), tumor necrosis factor-α (TNF-α), cyclooxygenase-2 (COX-2), and inducible NO synthase (iNOS). IL-1β, IL-6, and TNF-α act as the proinflammatory cytokines and immediately cause an inflammatory response in affected endometrium [24]. Furthermore, together with MCP-1, they act as chemokines. These chemokines are involved in the recruitment and activation of macrophages, neutrophils, eosinophils, basophils, monocytes, and natural killer cells (NK-cell) to the inflamed endometrium [32,33], whereas the NF-κB signaling pathway protects a survival of immune cells in the face of bacterial infections or irritation [34] (Figure 1).

### 2.3. Endometriosis

Endometriosis is a quite common benign gynecological human disease, affecting up to 50% of women with infertility [1]. In the course of endometriosis, the ectopic growth of the endometrium, predominantly glands, and stroma outside the uterus is observed [6]. The ectopic endometrium appears mainly in the ovaries and pelvic peritoneum, causing chronic pain, dysmenorrhea, and infertility [1]. 

**Figure 2:**
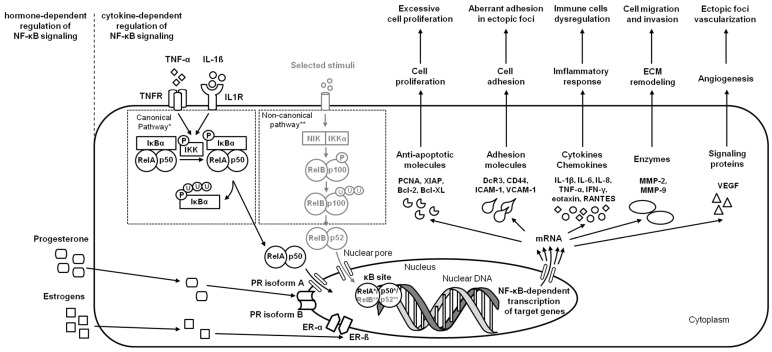
A simplified schema of the NF-κB signaling pathway in the endometrium in the case of endometriosis. Studied pathways are marked in black. Pathways requiring further research are marked in grey. Both canonical pathway * and noncanonical pathway ** are considered.

In response to multiple factors such as IL-1β, TNF-α [1], and TLR4 [19], endometriotic cells activate the NF-κB signaling pathway, affecting endometriosis initiation and progression. In the initial step of endometriosis, the NF-κB pathway induces upregulation of adhesion molecules, including Decoy receptor 3 (DcR3) [35], homing cell adhesion molecule (CD44), intercellular adhesion molecule 1 (ICAM-1), and vascular cell adhesion molecule-1 (VCAM-1) [36], which affect the aberrant adhesion of endometriotic cells in the ectopic locations [35]. On the progression step of endometriosis, the activation of the NF-κB pathway stimulates the release of IL-1β, IL-6, IL-8, TNF-α, interferon γ (IFN-γ), eotaxin, regulated on activation, normal T-cell expressed and secreted (RANTES) [6], and proliferating cell nuclear antigen (PCNA) [1], which promote a chronic inflammatory environment in endometriotic foci; enhances angiogenesis via increased production of vascular endothelial growth factor (VEGF) [6]; and avoidance of apoptosis via activation of the antiapoptotic molecules X-linked inhibitor of apoptosis (XIAP), B-cell lymphoma 2 protein (Bcl-2), and B-cell lymphoma-extra large protein (Bcl-XL) [1]. Moreover, the NF-κB pathway stimulates transcription of endopeptidases—matrix metalloproteinases (MMPs): MMP-2 and MMP-9; which are responsible for ECM degradation, and thus increasing endometriotic cell migration and invasion [20,36]. One may conclude that the ability of endometriotic cells to migrate and invade is crucial for the implantation and extension of the ectopic endometrium [1] (Figure 2).

### 2.4. Endometrosis

Endometrosis is a common benign gynecological equine disease, affecting up to 50% of older mares [30]. In the course of endometrosis, chronic degenerative changes in the endometrium are observed [28]. In affected endometrium, alterations of the epithelial and stromal cells lead to periglandular fibrosis and degeneration, dilatation, and atypical differentiation of the endometrial glands [30,37,38]. 

**Figure 3:**
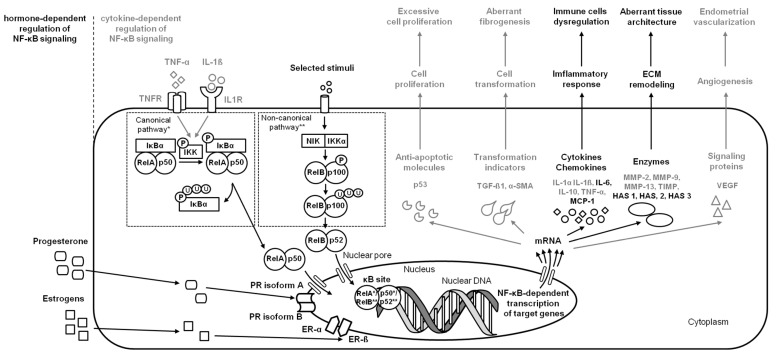
A simplified schema of the NF-κB signaling pathway in the endometrium in the case of endometrosis. Studied pathways are marked in black. Pathways requiring further research are marked in grey. Both canonical pathway * and noncanonical pathway ** are considered.

On the initial step of endometrosis, increased release of pro-fibrotic cytokines and chemokines (IL-1α, IL-1ß, IL-6, IL-10, and TNF-α) [39,40] stimulates immune cells infiltration and, via transforming growth factor- ß1 (TGF-ß1), up-regulates alpha smooth muscle actin (α-SMA) transcripts in resident fibroblasts, promoting their differentiation into myofibroblasts and thus leading to alternated secretion of ECM components and fibrogenesis [40,41,42]. On the next step of endometriosis, dysregulated immune cells synthesize profibrotic cytokines (IL-6, MCP-1, TNF-α, and TGF-ß1) that participate in remodeling of the endometrial ECM mediated by the modified activity of MMPs (up-regulated MMP-2 and MMP-9 transcription and downregulated MMP-13 transcription), decrease the activity of the tissue inhibitors of metalloproteinases (TIMP) [40,41,42,43,44], and increase activity of hyaluronan synthases (HASs) [8,9]. One may observe that the cascade of endometrial changes in endometrosis is similar to those described in other endometrial diseases; however, the knowledge of the role of NF-κB in its signaling pathway is limited. The similarities found in the signs of immune cell dysregulation and ECM remodeling [8,40,41,42] and the genetic background [45] of equine endometriosis with other endometrial diseases provide a rationale for further in-depth investigations. So far, with the increased progression of endometrosis, the increased activation of canonical (RelA/NF-κB1) and noncanonical (RelB/NF-κB2) signaling pathways, resulting in downregulation of IL-6 releasing and up-regulation of HAS 1 and HAS 3 activity, were demonstrated [8]. Moreover, the increased activation of canonical (RelA/NF-κB1) signaling pathways resulted in up-regulation of IL-6 and MCP-1 release as well as up-regulation of HAS 2 activity, which was noted in destructive histopathological types of endometrosis [9]. While other potentially relevant receptors, enzymes, cytokines, chemokines, or other signaling proteins have not yet been studied in the context of the NF-κB signaling pathway, which does not rule out their involvement in the pathogenesis of endometrosis, the need for further study of NF-κB signaling in endometrosis is indicated by the results of the latest research, according to which advanced endometrial fibrosis may be transformed into cancer [45]. In the case of endometrosis, changes in the expression of both metabolism-related genes (*CYP1B1*, *COX4I1*, *COX3*, and *UQCRFS1*) and immune response genes (*MMP7*, *JCHAIN*, *PIGR*, *CALR*, *B2M*, and *FCGRT*) may alter the metabolic-immune microenvironment in affected endometrium, predisposing fibrotic tissue to carcinogenesis [45]. Especially since ECM deposition, remodeling, and cross-linking driving fibrosis to stiffen the endometrial stroma are characteristic of human endometrial carcinoma [21,23,46] (Figure 3).

### 2.5. Endometrial Carcinoma

Endometrial carcinoma is one of the most common forms of malignant gynecological disease in humans [47], and at the same time the second most common gynecological neoplasm and the fourth most frequent cancer in women worldwide [22]. In contrast to women, endometrial carcinoma is rarely reported as single case reports in mares [48,49,50,51,52], and even less often in cattle [53].

**Figure 4:**
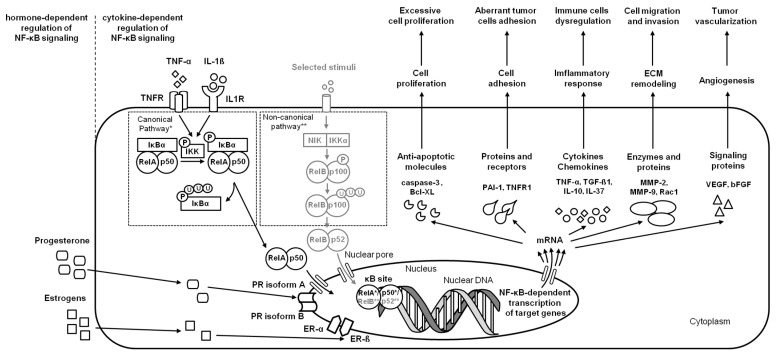
A simplified schema of the NF-κB signaling pathway in the endometrium in the case of endometrial carcinoma. Studied pathways are marked in black. Pathways requiring further research are marked in grey. Both canonical pathway * and noncanonical pathway ** are considered.

In the course of endometrial carcinoma, the two main clinico-pathological variants of endometrial carcinoma are considered—type 1 representing low-grade and estrogen-dependent endometrioid carcinomas, and type 2 representing high-grade, aggressive, and estrogen-independent nonendometrioid carcinomas [54]. Type 1 usually coexists with or is preceded by endometrial hyperplasia, whereas type 2 arises occasionally in endometrial polyps or from precancerous lesions developing in atrophic endometrium [16]. Both types of carcinogenesis are related to the NF-κB signaling pathway by the regulation of genes expression involved in apoptosis, the cell cycle, differentiation, and cell migration. The activity of NF-κB pathways may provide a survival advantage for tumor cells by the suppression of apoptosis via regulation of target genes Bcl-XL [16] and caspase-3 (CASP3) [47]. Moreover, the activity of NF-κB in endometrial carcinoma promotes tumor cell migration, invasion, and metastasis via stimulation of Ras-related C3 botulinum toxin substrate 1 (Rac1), MMP-2 [24], and MMP-9 [21,23] transcription, and thus the basement membranes’ and ECM degradation, respectively [21,23]. Interestingly, in endometrial cancer cells both Rac1—an indicator of tumor cell migration and invasion, and MMP-2—an indicator of tumor metastasis and primary tumor growth—were downregulated by IL-37 [24]. IL-37 is an anti-inflammatory cytokine [55] involved in immune responses [56] and tumorigenesis [57] through the downregulation of pro-inflammatory cytokines and inhibition of metastasis, respectively. Moreover, the activity of NF-κB pathways may also decrease the expression of IL-37 protein in cancer cells, confirming the inhibitory roles of IL-37 on metastasis in endometrial carcinoma [23] (Figure 4).

## 3. Regulation of NF-κB Signaling in Endometrium

It has been evidenced that the hormone-dependent immune system dysregulation in response to the aberrant production of chemokines and cytokines plays a role in the initiation and progression of endometrial diseases, such as endometritis [7,17], endometriosis [1,6], endometrosis [11], and endometrial carcinoma [23]. In both humans and animals, hormonal therapies, based on the creation of hypoestrogenic (GnRH agonists), hyperandrogenic (danazol and gestrinone), or hyperprogestogenic (progesterone and progestins) environments are commonly used [6,58,59], often causing systemic side effects due to the suppression of endometrial cell proliferation and endogenous steroid hormone concentrations [6,60]. Therefore, the hormone-dependent regulation of NF-κB signaling in the endometrium is summarized here to highlight the hormone-NF-κB interaction.

### 3.1. Progesterone’s Regulation

In both, humans [61,62] and livestock animals [7], the uterus is resistant to infections with decreasing blood progesterone concentration and is susceptible to infections with increasing blood progesterone concentration. In human endometrium, progesterone was proven to inhibit TLR4 expression, NF-κB activation, as well as IL-6 and IL-8 productions, limiting the effectiveness of inflammation in response to bacterial infection [17]. Similarly, in bovine endometrium, progesterone was found to decrease the expressions of IL-1β, IL-6, IL-8, and TNF-α via the NF-κB and MAPK signaling pathways [7]. The progesterone-related suppression of the NF-κB pathway was observed in endometrial cells with high progesterone receptors (PR) expression, contrary to weak PR expression, suggesting the PR-mediated action [17]. Moreover, it has been proven that NF-κB (RelA (p65)) and PR can repress each other through direct contact [63]. 

Progesterone has two isoforms of PRs (PR isoform A and PR isoform B) encoded by the same gene, whereas PR isoform A is predominant in the uterus and ovaries [64,65]. In endometrial epithelium and stroma, progesterone passes through the cells and binds to an intranuclear receptor, then regulates cell development and differentiation by inducing multiple genes transcription [65,66]. Progesterone binds to PR isoform A and acts as an antagonist for estrogen-induced epithelial cell proliferation. Moreover, progesterone downregulates PR expression, and thus regulates both its biological effects and receptor abundance [30,67]. In human endometrium, these biological alterations during the ovarian cycle are also regulated via NF-κB (RelA (p65))/PR repression [68]; however, in animals, the NF-κB-PR interaction in the ovarian cycle requires confirmation. So far, in equine endometrium, no correlations were found between genes expression of RelA (p65), NF-kB1, NF-kB2, and PR in both the follicular and luteal phases of the ovarian cycle [11]. In human endometrium, the NF-κB-PR interaction was suggested to be involved in pathophysiologic processes, such as irregular uterine bleeding [69] and endometriosis [1,70,71]. In the case of recurrence of ovarian endometriosis, both inverse [70] and direct [71] relations between RelA (p65) and PR were reported; however, both researchers considered PR isoform B as predominant [70,71]. In equine endometrium, the initial signs of NF-κB-PR interaction were indicated in mild endometrosis by the strong positive correlation between RelA and PR genes transcription [11]. In the case of endometrial carcinoma, progesterone exertion was suspected to inhibit anti-inflammatory cytokines production, which was confirmed for IL-10 [62] and excluded for IL-37 [23]. The recent result suggests some signs of hormone-dependent immune system dysregulation in endometrial carcinogenesis. However, regarding the role of the NF-κB-PR interaction, this requires further research.

### 3.2. Estrogen’s Regulation

Contrary to the blood progesterone concentration in both humans [61,62] and livestock animals [7], the uterus is susceptible to infections with the decreasing of blood estrogen concentration and is resistant to infections with the increasing of blood estrogen concentration. In endometrial epithelium and stroma, estrogen passes through the cells, binds to an intranuclear receptor, and regulates cell development and differentiation by inducing multiple genes transcription [65,66]. In the canonical pathway, estrogen has two specific estrogen receptors (ERs) (ER-α and ER-ß) encoded by different genes. ER-α is predominant in the uterus [65,72]. The binding of estradiol to ER-α and ER-ß has contradictory uterotrophic effects. The activation of ER-α stimulates the proliferation of the epithelial cells and stromal cells, whereas the activation of ER- ß inhibits it. Moreover, the activation of ER-α upregulates PR expression, whereas the activation of ER- ß downregulates it [30,67]. In healthy human endometrium, the negative crosstalk between ER-α and NF-κB was confirmed as a part of the regulatory process of normal physiological responses [73]. In horses, the NF-κB-ER-α interaction has not been confirmed so far, since no correlations were found between genes expression of RelA (p65), NF-kB1, NF-kB2, and ER-α and ER-ß in both phases of the ovarian cycle [18]. Considering endometriosis as an estrogen-dependent disease, the NF-κB-ER interaction was suggested to be involved in its pathogenesis. It can be observed that estrogen promotes the implantation of endometrial tissue to ectopic foci, and thus is necessary but not sufficient for sustaining endometriosis [20]. In the ectopic endometrial cells, ER-α and ER-β were reported as able to increase NF-κB activity [74] by activating several proinflammatory pathways (CXCL12/CXCR4, PI3K/Akt), and thus promote the viability and proliferation of endometriotic cells [75]. In other studies on endometriosis, the inhibitory effect of estrogen signaling on NF-κB has been reported [76,77]. Estrogen was able to reduce the expression of a gene that encodes the angiotensin II receptor (AGTR1), and thus activates NF-κB signaling [77]. Moreover, in the eutopic endometrium, ER-β was able to downregulate TNFα/NF-κB signaling [76]. Therefore, the effects of estrogen signaling on NF-κB in endometriosis are controversial [1]. In equine endometrium, the initial signs of NF-κB-ER interaction were indicated in inactive nondestructive, active nondestructive, and active destructive types of endometrosis by moderate to strong negative correlations between RelA, NF-kB1, and ER-β genes transcription [11]. In the case of endometrial carcinoma, estradiol action was suspected to inhibit IL-10 [62], but not IL-37 [23] anti-inflammatory cytokines production. Moreover, in the endometrial carcinoma model, the NF-κB activity was induced by different concentrations of estradiol in a rapid, non-genomic, and non-receptor manner. It was confirmed that activation of NF-κB plays a role in estradiol-induced angiogenesis by up-regulator basic fibroblast growth factor (bFGF) and VEGF, a major angiogenic factor that induces endothelial cell proliferation and thus promotes tumor-induced angiogenesis [78]. Therefore, the role of the NF-κB–estrogen interaction, both receptor and non-receptor, in the hormone-dependent immune system dysregulation in endometrial diseases requires further research.

## 4. Perspectives for NF-κB Signaling in Endometrium

Over 700 compounds were identified as inhibitors of the NF-κB pathway, used to decrease transcription of genes [1] of several receptors (TLR4, ER-α, ER-ß, PGR, and TNFR1), enzymes (COX-2, iNOS, MMP-2, MMP-9, HAS 1, HAS 2, and HAS 3), cytokines/chemokines (IL-1α, IL-1β, IL-4, IL-6, IL-7, IL-8, IL-13, IL-15, IL-17, IL-18, IL-37, MCP-1, TNF-α, TGF-ß1, eotaxin, INF-γ, IP-10, MIP-1α, MIP-1β, and RANTES), and other signaling proteins (FGF, G-CSF, VEGF, Bcl-XL, caspase-3, PAI-1, and Rac1), respectively [6,7,8,9,15,16,17,18,19,20,21,22,23,24].

The biggest interest regarding influencing NF-κB signaling regards cancer treatment. In human medicine plenty of compounds of different origin have been found to inhibit this pathway; however, they are still in development and research. Most studies are conducted in isolated endometrial cells, cancer cells, or in mice models, hence clinical effectiveness is yet unknown.

Dehydroxymethylepoxyquinomicin (DHMEQ) is a specific inhibitor of NF-κB which binds to Rel proteins. It was found to induce apoptosis, stop cell cycles, and decrease proinflammatory cytokines secretion in cancer cell lines [79].

Mushroom-produced compounds are widely evaluated in the context of cancer therapy. Polysaccharide produced by *Ganoderma lucidum* is capable of inhibiting classical activation of NF-κB, which decreases P-glycoprotein expression in cancer cells. P-glycoprotein is one of the factors responsible for drug resistance, as it decreases transport of chemotherapeutic agents. In that way, this polysaccharide enables the effectiveness of anticancer drugs. Another polysaccharide decreasing levels of NF-κB is produced by *Phellinus linteus.* This species produces another compound, caffeic acid phenethyl ester (CAPE), which inhibites NF-κB binding to DNA. Another mechanism influencing NF-κB activation is promoting TNF-α, and thus suppressing anti-apoptotic action of NF-κB, which can be achieved with cordycepin produced by *Cordyceps militaris*, a parasitic fungus [2].

Another group of active substances is produced by plants. Parthenolide, acquired from feverfew (*Tanacetum parthenium*) was proven to inhibit NF-κB both in human and canine cancer cells [80]. Curcumin is already a quite well-known compound with a plethora of biological activities. It was found that in endometrial cancer liposomal curcumin decreased NF-κB expression in correlation with curcumin concentration [21].

Curcumin was also found to repress activation of NF-κB induced by TNF-α and IL-1β in endometriosis, with effects such as reduced production of proinflammatory cytokines, chemokines, and cell adhesion molecules. Other plant extracts were also evaluated in the context of endometrosis, such as andrographolide from *Andrographis paniculate*. Its mechanism is to inhibit NF-κB binding with DNA, and thus decrease the proliferation of endometriotic cells, resulting in lesion size reduction. Another compound with anti-inflammatory action in endometriosis is astragaloside IV, produced by *Astragalus membranaceus* by inhibition of the TLR4/NF-κB pathway [1,22].

Synthetic compound BAY 11-7085 prevents the phosphorylation of IκBα and release of NF-κB. A result of this is increased endometriotic cell apoptosis and reduced proliferation. To a similar effect, suppression of IκBα phosphorylation can be achieved with pyrrolidine dithiocarbamate, a chelating agent. Endometriotic cell apoptosis can also be induced with BV6, an antagonist of inhibitors of apoptosis proteins. On the other hand, thalidomide and pioglitazone, apart from their main mechanisms of action, can also decrease IL-8 secretion by inhibition of NF-κB activation by TNF-α in endometriosis [1].

Reverbα is a gene responsible for the circadian clock. Its activation inhibits activation of NF-κB by LPS, giving future perspectives for endometritis treatment. However, currently there is not much interest in NF-κB inhibition in endometritis. In endometrosis in mares NF-κB influence is only being evaluated; however, some results suggest that in future it can be considered as a therapeutic target [20].

Despite the conducted research, NF-κB inhibitors are still under development as treatment for endometrial diseases. As there is huge variability in active substances and their mechanisms of action it is extremely challenging to develop efficient and safe drugs. Proving the biological effect of a substance is an obvious initial step, but because of variety of the pathways activated by NF-κB, sometimes with contradictory actions, other possible consequences of NF-κB inhibition have to established. It is important to evaluate if NF-κB inhibition would be beneficial considering its role in cellular senescence. Usually it is safer to use more specific drugs, affecting a narrower range of cell and tissue physiology [1,78,81,82].

As NF-kB is composed of the same proteins in mammals, animal models can be relatively easily adapted for research and clinical trials. Additionally, it gives the opportunity to transfer data and knowledge between species. However, it is important to exclude compound activity in other physiological pathways, which would cause side effects [3,78,81]. Clinical trials are an important next step in future studies, even using animal models more closely related to humans than mice. On the other hand, animal species are still a step behind regarding drug development, and more fundamental research has yet to be conducted [4].

## 5. Conclusions

The aberrant NF-κB signaling pathway, causing immune system dysregulation, is involved in several aspects of endometrial disease pathogenesis in both humans and animals. This dysregulation in response to the aberrant production of chemokines and cytokines plays a role in the initiation and progression of endometritis, endometriosis, endometrosis, and endometrial carcinoma. Moreover, this immune system dysregulation seems to be both NF-κB-dependent and hormone-dependent; therefore, future studies should be focused on the hormone-dependent regulation of NF-κB signaling in the endometrium, as the detailed identification of hormone–NF-κB interaction is an important step toward understanding the pathogenesis of these conditions and developing effective strategies for the treatment of common endometrial diseases. Future perspectives in studies regarding the NF-κB pathway in endometrial diseases regard performing clinical trials for the development of treatment.

## Figures and Tables

**Table 1:** The selected recent research on pathways, targets, and mechanisms of the most common endometrial diseases (endometritis, endometriosis, endometrosis, and endometrial carcinoma) was carried out on humans and animals (cattle, mouse, and horse) specimens.

Disease	Species	Tissue	Pathways	Targets/Mechanisms	Reference
endometritis	Human	hEEC	NF-κB	increase TLR4, IL-6, IL-8, and MCP-1 expression	[13]
endometritis	Cattle	BEEC	NF-κB (RelA (p65), IκBα) and MAPK	increase IL-1β, IL-6, IL-8, TNF-α, COX-2, and iNOS expression	[14]
endometritis	Human	hESC	NF-κB (pNF-κB, NF-κB)	increase IL-1β, IL-6, IL-8, IL-18, and TNFαexpression	[15]
endometritis	Cattle	BEEC	NF-κB (RelA (p65), IκBα) and MAPK	increase IL-1β, IL-6, IL-8, and TNF-α expression	[4]
endometriosis	Human	hESC	NF-κB (pNF-κB, NF-κB, pIKKβ, IKKβ, pIKKα, IKKα), STAT3 and JNK	increase IL-1α, IL-1β, IL-4, IL-6, IL-7, IL-8, IL-13, IL-15, IL-17, eotaxin, FGF, G-CSF, INF-γ, IP-10, MCP-1, MIP-1α, MIP-1β, RANTES, TNF-α, and VEGF expression	[3]
endometriosis	Human	endometrium	NF-κB (RelA (p65))	increase MMP-2 and MMP-9 expression	[16]
endometriosis	Mouse Model	endometrium	NF-κB (RelA (p65))	increase IL-1β, IL-6, MCP-1, and TNF-α expression	[17]
endometrosis	Horses	endometrium	NF-κB (RelA (p65), NF-κB1 (p50/p105), NF-κB2 (p52/p100))	decrease IL-6 and increase HAS 1 and HAS 3 expression	[5]
endometrosis	Horses	endometrium	NF-κB (RelA (p65), NF-κB1 (p50/p105))	increase IL-6, MCP-1, and HAS 2 expression	[6]
endometrosis	Horses	endometrium	NF-κB (RelA (p65), NF-κB1 (p50/p105), NF-κB2 (p52/p100))	decrease ER-α, ER-ß, and PR expression	[18]
endometrial carcinoma	Human	endometrium	NF-κB (RelA (p65), NF-κB1 (p50/p105), NF-κB2 (p52/p100), cRel, RelB, IκBα, IκBβ, IκBε, and Bcl-3)	increase Bcl-XLexpression	[12]
endometrial carcinoma	Human	hECC	NF-κB (RelA (p65))	decrease caspase-3 activation and increase MMP-9 expression	[19]
endometrial carcinoma	Human	endometrium	NF-κB (RelA (p65), pRelA (p65), IκBα)	increase TNF-α, PAI-1, TGF-ß1, and TNFR1 expression	[20]
endometrial carcinoma	Human	endometrium, hECC	NF-κB (pIKKα, pIKKβ, IκBα), JNK, and MAPK	decrease IL-37 expression and increase MCP-2, MCP-9, and Rac1 expression	[24]

hEEC—human endometrial epithelial cells; BEEC—bovine endometrial epithelial cells; hESC—human endometrial stromal cells; hECC—human endometrial carcinoma cells; NF-κB—nuclear factor κ-light-chain-enhancer of activated B cells; RelA (p65)—protein RelA of NF-κB superfamily; pRelA (p65)—phospho-protein RelA of NF-κB superfamily; NF-κB1 (p50/p105)—protein NF-κB1 of NF-κB superfamily; NF-κB2 (p52/p100)—protein NF-κB2 of NF-κB superfamily; cRel—protein cRel of NF-κB superfamily; RelB—protein RelB of NF-κB superfamily; IκB—family of inhibitors of κB; pNF-κB—phospho-nuclear factor κ-light-chain-enhancer of activated B cells; pIKKβ—phospho-inhibitor of nuclear factor κ-B kinase subunit β; IKKβ—inhibitor of nuclear factor κ-B kinase subunit β; pIKKα—phospho-inhibitor of nuclear factor κ-a kinase subunit α; IKKα—inhibitor of nuclear factor κ-a kinase subunit β; MAPK—mitogen-activated protein kinase; STAT3—activator of transcription 3; JNK—c-Jun N-terminal kinases; TLR4 – Toll-like receptor 4; IL—interleukin; MCP-1/CCL2—monocyte chemotactic protein-1; TNF-α—tumor necrosis factor-α; COX-2—cyclooxygenase-2; iNOS—inducible NO synthase; FGF—fibroblast growth factors; G-CSF—granulocyte-colony stimulating factor; GM-CSF—granulocyte-macrophage colony stimulating factor; IFNγ—interferon γ; IP-10/CXCL10—interferon γ-induced protein 10; MIP-1α/CCL3—macrophage inflammatory proteins 1α; MIP-1β/CCL4—macrophage inflammatory proteins 1β; RANES/CCL5—regulated on activation, normal T-cell expressed and secreted; VEGF—vascular permeability factor/vascular endothelial growth factor; MMP—matrix metalloproteinases; HAS—hyaluronan synthase; ER—estrogen receptor; PR—progesterone receptors; Bcl-XL—B-cell lymphoma-extra large; TNFR1—tumor necrosis factor receptor 1; TGF-ß1—transforming growth factor- ß1; PAI-1—plasminogen activator inhibitor 1.

## Data Availability

The data presented in this study are available on request from the corresponding author.
